# Supplementary material for: Conversion of Osteoclasts into Bone-Protective, Tumor-Suppressing Cells
Source: Cancers (Basel). 2021 Nov 9;13(22):5593. doi: 10.3390/cancers13225593 (PMC8615769; doi:10.3390/cancers13225593)
Supplement: Supplementary file 1 [file cancers-13-05593-s001.zip › cancers-1415908-supplementary.pdf]

Supplementary Materials

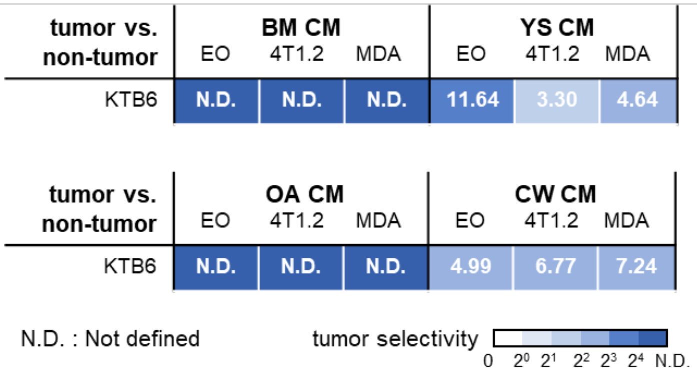

Suppl. Figure S1. Selective inhibition of the MTT-based viability of 3 tumor cell lines (EO = EO771 mammary tumor cells, 4T1.2 = 4T1.2 mammary tumor cells, and MDA = MDA-MB-231 breast cancer cells) by BM CM, YS CM, OA CM, and CW CM, compared to human breast epithelial cells (KTB6). BM = BML284 (Wnt activator), YS = YS49 (PI3K/Akt activator), OA = OAC2 (Oct4 activator), and CW = CW008 (PKA activator). Of note, N.D. means "not defined," in which non-tumor cells increased their MTT-based viability.

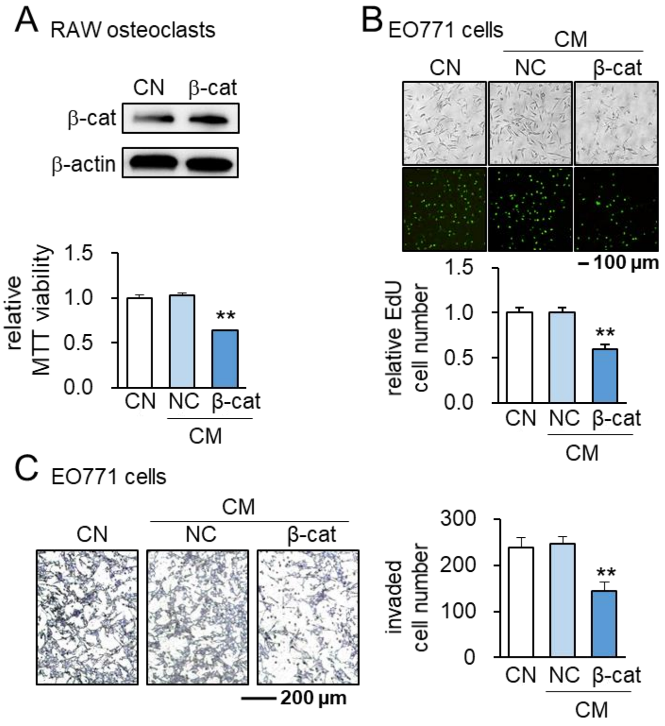

Suppl. Figure S2. Tumor-suppressive effects of β-catenin overexpressing CM derived from RAW cells. CN = control, NC = negative control, CM= RAW264.7 osteoclasts-derived CM, and β-cat = β-catenin overexpression. The double asterisk indicates $p < 0.01$. (A-B) Suppression of MTT-based viability, and EdU-based proliferation of EO771 tumor cells by β-catenin- overexpressing RAW cell-derived CM. (C) The reduction in transwell invasion of EO771 tumor cells by β-catenin CM.

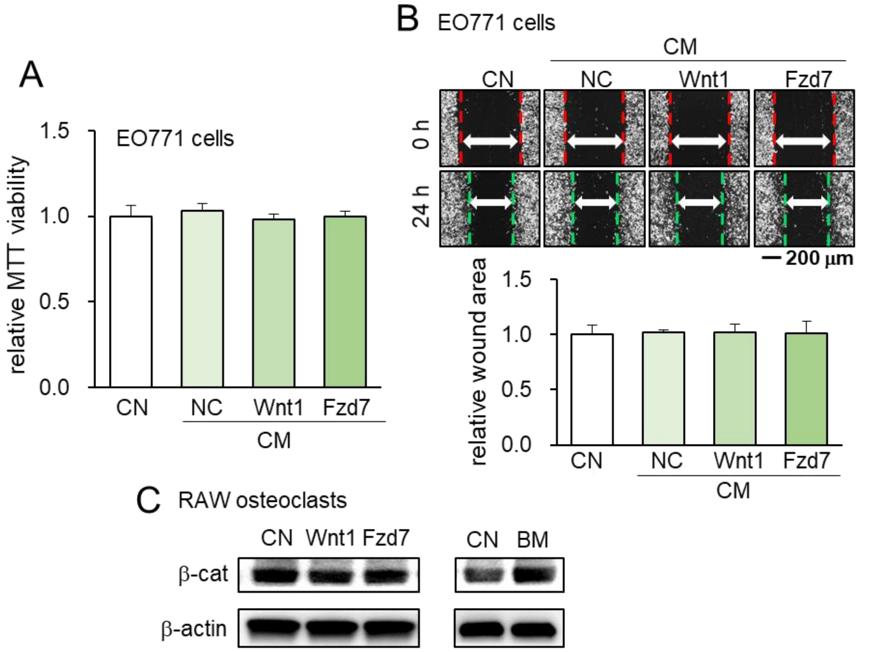

Suppl. Figure S3. Wnt1 and Fzd7 as a negative control for the induction of tumor-suppressive secretomes. CN = control, NC = negative control, β-cat = β-catenin, BM= BML284, CM= RAW264.7 osteoclasts-derived CM, Wnt1 = Wnt1 overexpression, and Fzd7 = Fzd7 overexpression. (A&B) No detectable changes by the overexpression of Wnt1 and Fzd7 in RAW264.7 osteoclasts in MTT-based viability and scratch-based motility, respectively, in EO771 mammary tumor cells. (C) Unchanged level of β-catenin by the overexpression of Wnt1 and Fzd7, and its elevation by the treatment with BML284 in RAW264.7 osteoclasts.

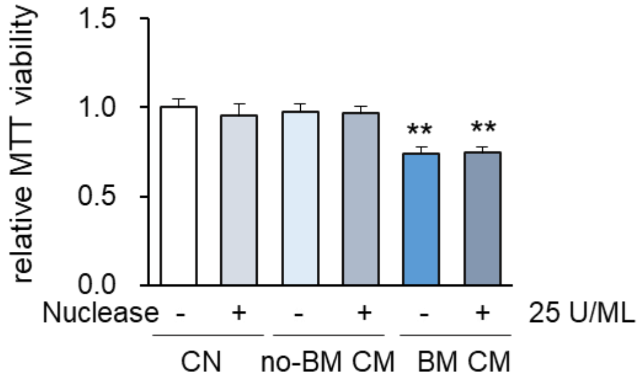

Suppl. Figure S4. No detectable effect of nuclease-treated BM CM on MTT- based viability of EO771 tumor cells. no-BM CM = without BML284-treated RAW-derived conditioned medium, and BM CM = BML284-treated RAW- derived conditioned medium. The double asterisk indicates $p < 0.01$. The result suggests that anti-tumor ability is not induced by nucleic acids such as DNA and RNA.

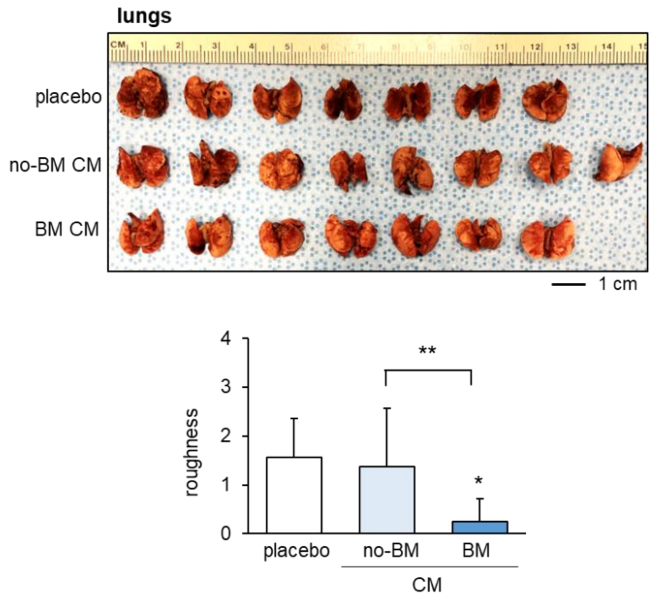

Suppl. Figure S5. Reduction in the damage score of the lung surface by the daily administration of BM CM for 3 weeks. The damage score was defined as 0 = normal, 1 = minor damage, 2 = moderate damage, and 3 = significant damage. The single and double asterisks in the figures indicate $p < 0.05$ and 0.01, respectively.

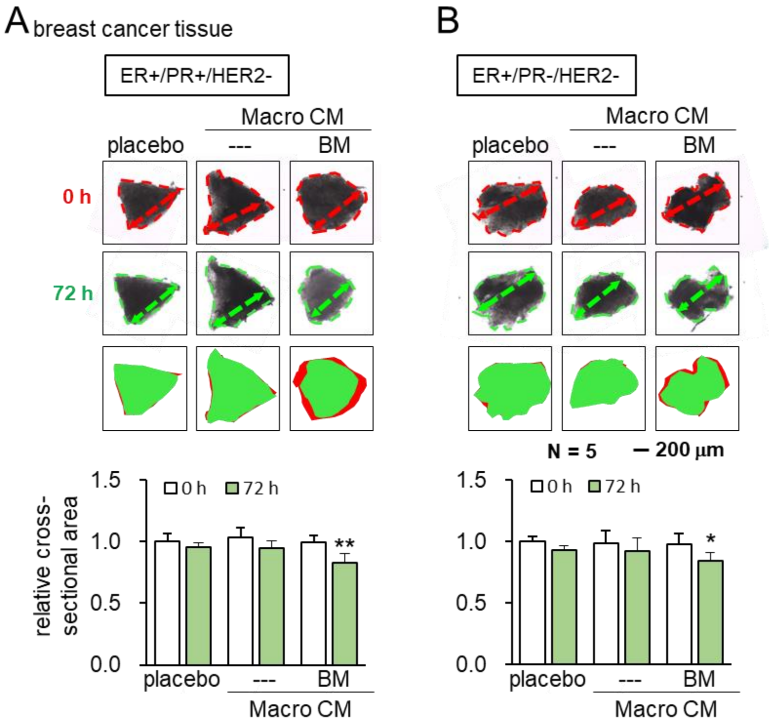

Suppl. Figure S6. Shrinkage of human breast cancer tissue fragments by BML284-treated human macrophage-derived CM. Macro CM = human macrophage-derived conditioned medium, and BM = BML284 treatment. The single and double asterisks in the figures indicate $p < 0.05$ and 0.01, respectively.

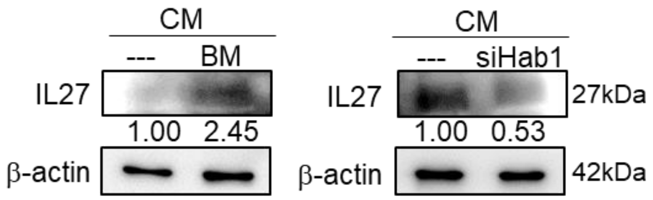

Suppl. Figure S7. Elevation of a tumor-suppressive cytokine, IL27, in BM CM, and its reduction in Hsp90ab1 siRNA-treated RAW osteoclasts-derived CM.

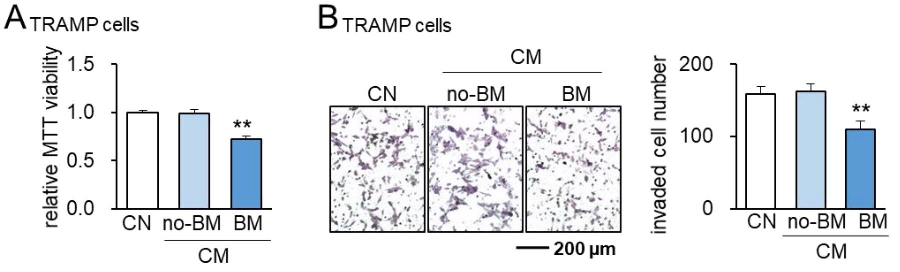

Suppl. Figure S8. Responses of TRAMP-C2ras prostate tumor cells to BM CM. CN = control, CM = RAW264.7 osteoclasts-derived conditioned medium, no-BM = without BML284 treatment, and BM = BML284 treatment. The double asterisk indicates $p < 0.01$. (A&B) Reduction in MTT-based viability and transwell invasion.

Suppl. Figure S9. Uncropped gels

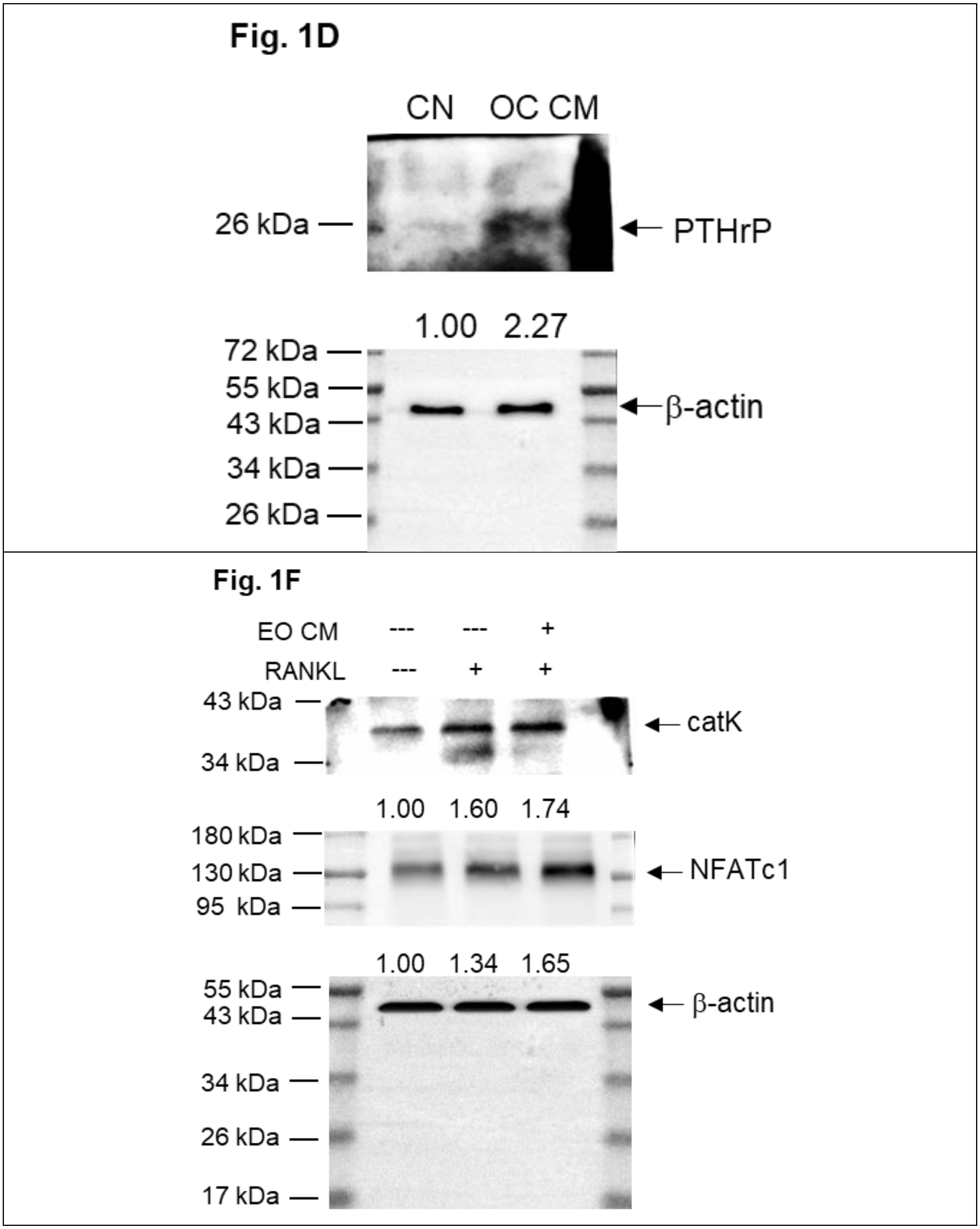

Fig. 2G

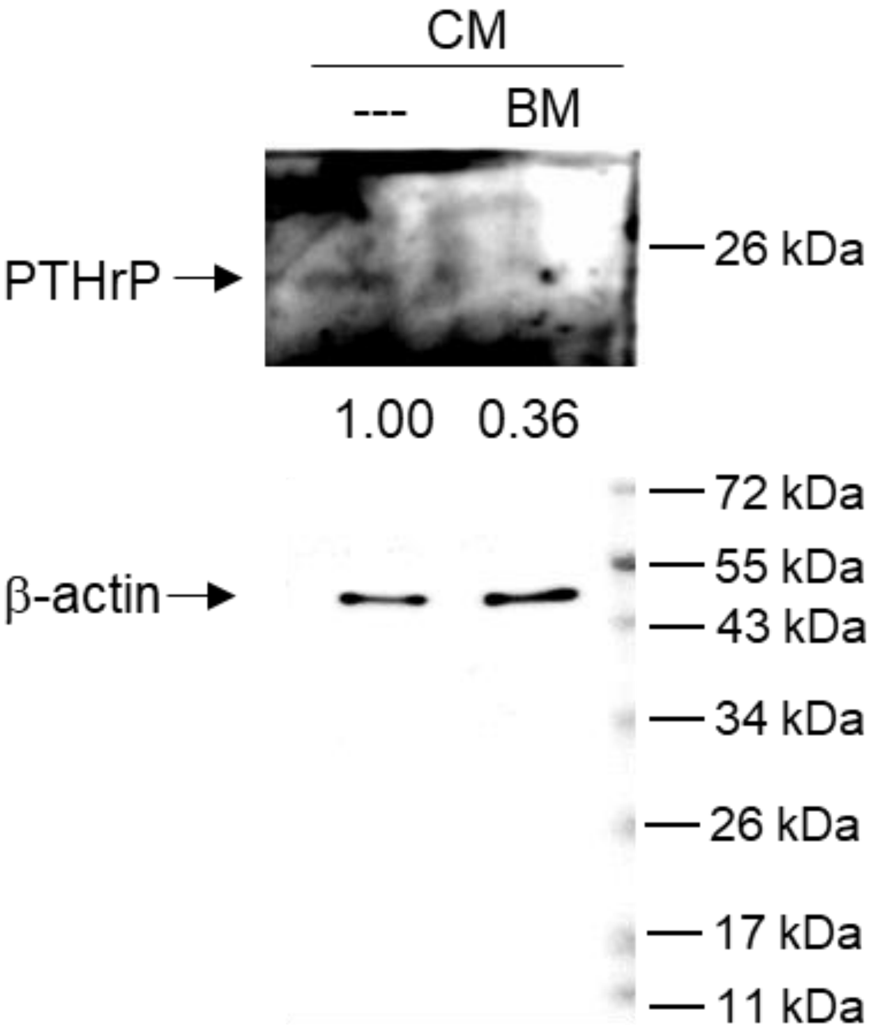
CM

BM
PTHrP
26 kDa
1.00
0.36
72 kDa
55 kDa
β-actin
43 kDa
34 kDa
26 kDa
17 kDa
11 kDa

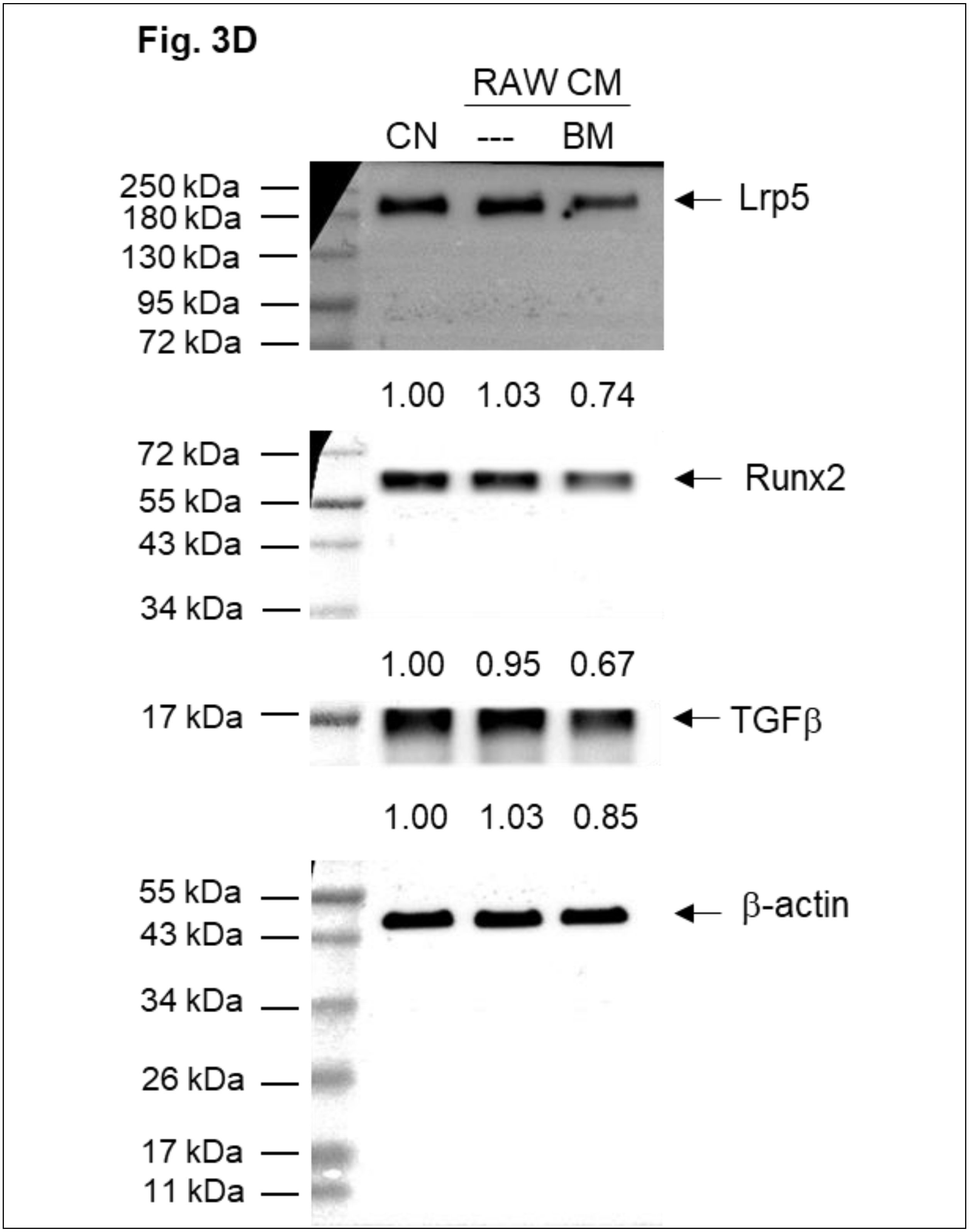
Fig. 3D
RAW CM
CN

BM
250 kDa
180 kDa
130 kDa
95 kDa
72 kDa
Lrp5
1.00
1.03
0.74
72 kDa
55 kDa
43 kDa
34 kDa
Runx2
1.00
0.95
0.67
17 kDa
TGFβ
1.00
1.03
0.85
55 kDa
43 kDa
34 kDa
26 kDa
17 kDa
11 kDa
β-actin

Fig. 6B

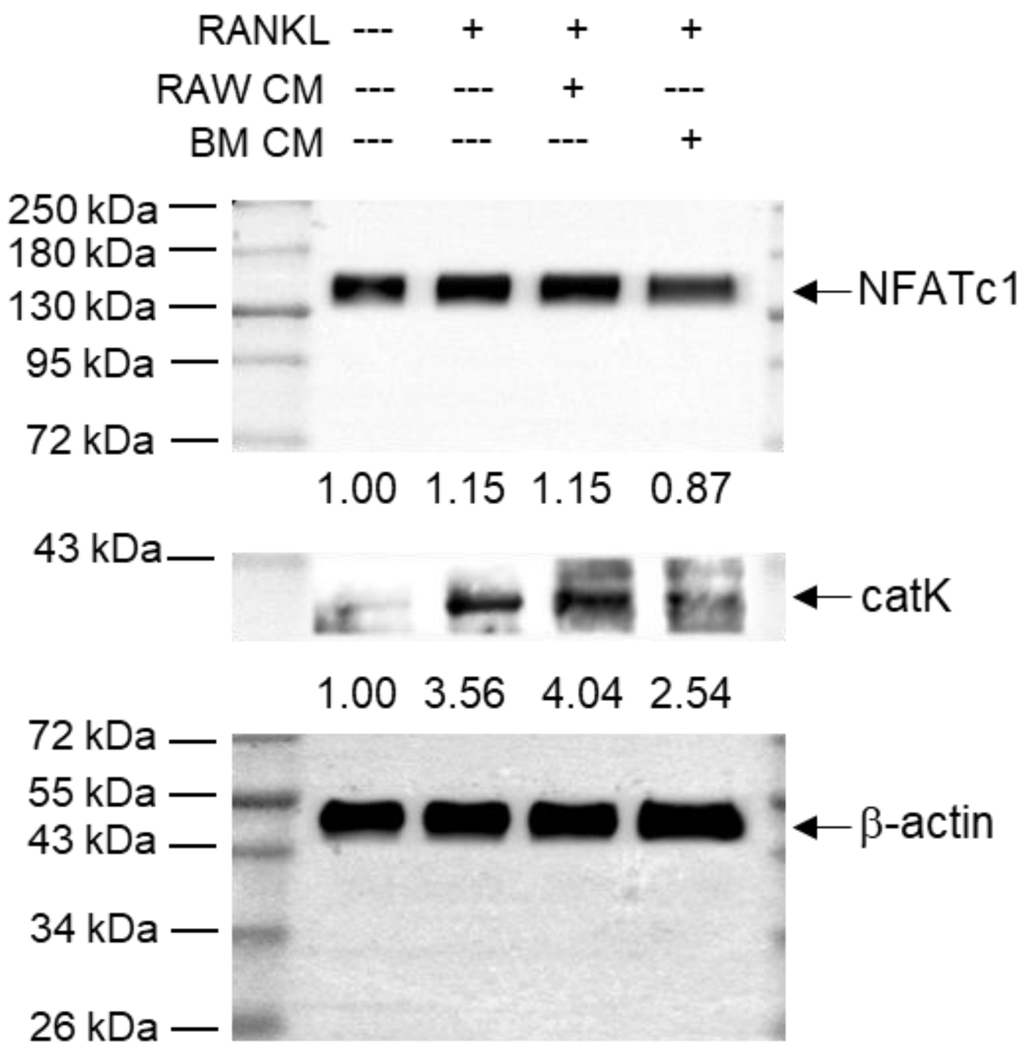

RANKL --- + + +
RAW CM --- --- + ---
BM CM --- --- --- +
250 kDa
180 kDa
130 kDa
95 kDa
72 kDa
NFATc1
1.00 1.15 1.15 0.87
43 kDa
catK
1.00 3.56 4.04 2.54
72 kDa
55 kDa
43 kDa
34 kDa
26 kDa
β-actin

Fig. 6D

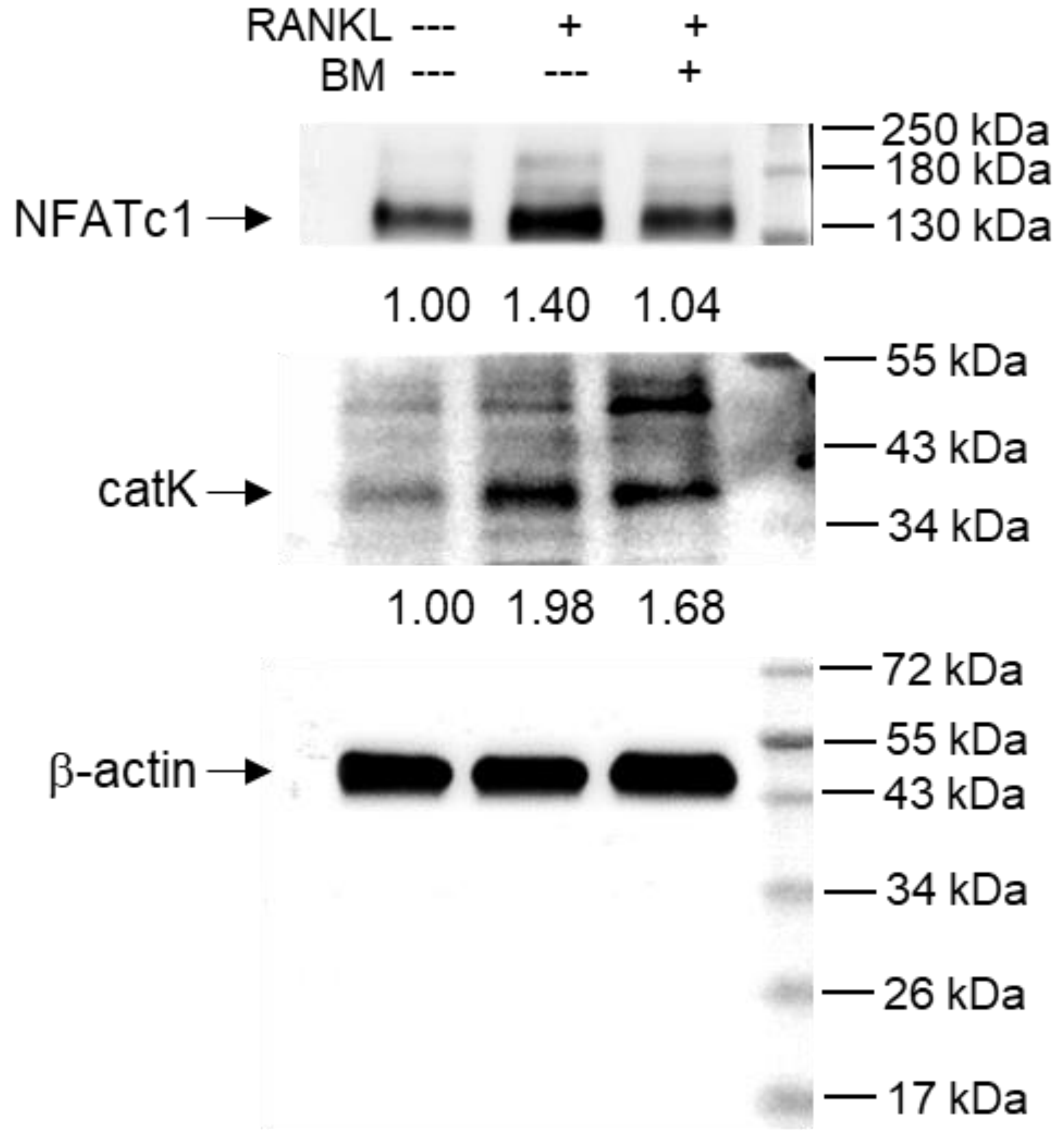

RANKL --- + +
BM --- --- +
NFATc1
250 kDa
180 kDa
130 kDa
1.00 1.40 1.04
catK
55 kDa
43 kDa
34 kDa
1.00 1.98 1.68
β-actin
72 kDa
55 kDa
43 kDa
34 kDa
26 kDa
17 kDa

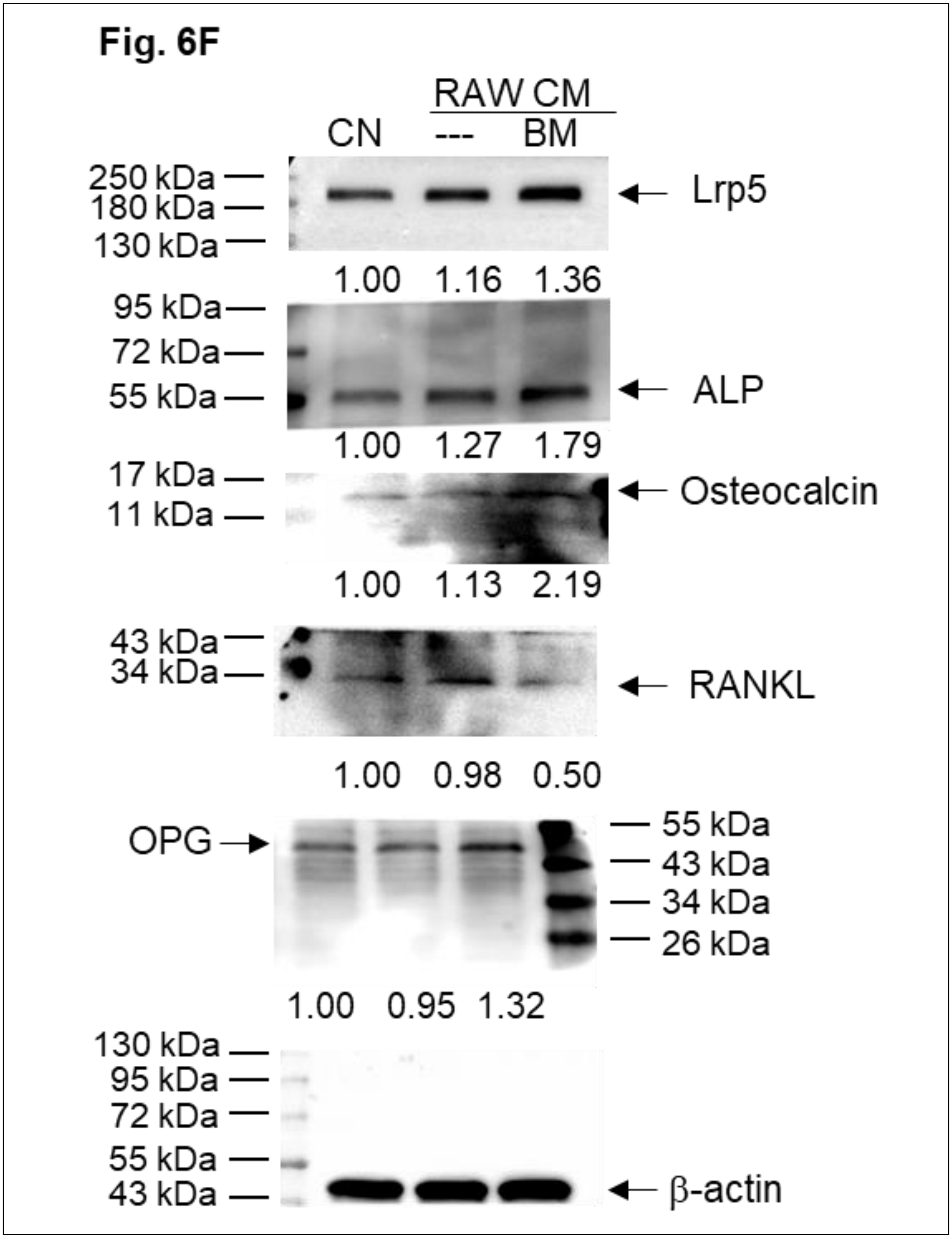
Fig. 6F
RAW CM
CN

BM
250 kDa
180 kDa
130 kDa
Lrp5
1.00 1.16 1.36
95 kDa
72 kDa
55 kDa
ALP
1.00 1.27 1.79
17 kDa
11 kDa
Osteocalcin
1.00 1.13 2.19
43 kDa
34 kDa
RANKL
1.00 0.98 0.50
OPG
55 kDa
43 kDa
34 kDa
26 kDa
1.00 0.95 1.32
130 kDa
95 kDa
72 kDa
55 kDa
43 kDa
β-actin

Fig. 6G

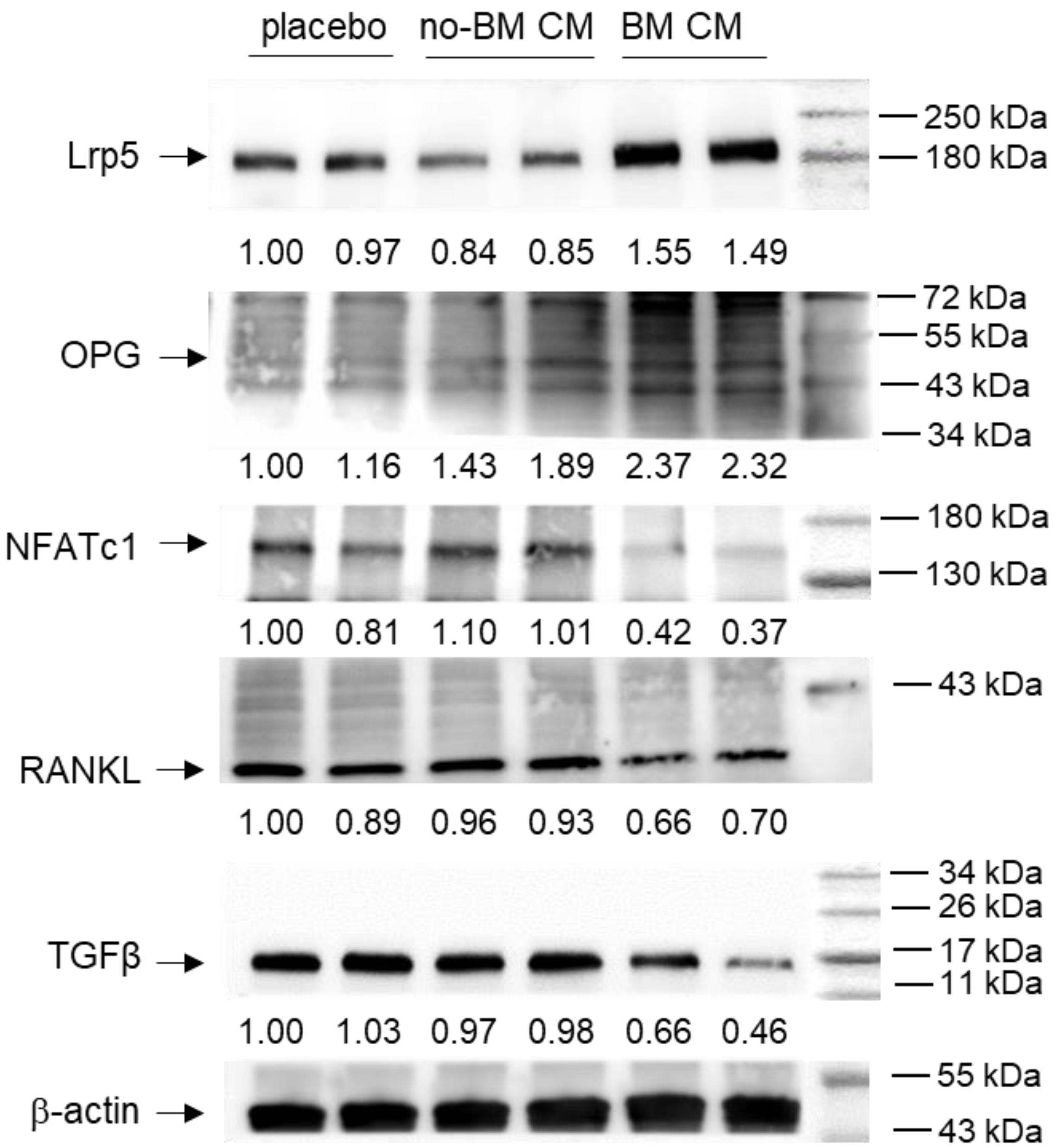

placebo
no-BM CM
BM CM
Lrp5
250 kDa
180 kDa
1.00 0.97 0.84 0.85 1.55 1.49
OPG
72 kDa
55 kDa
43 kDa
34 kDa
1.00 1.16 1.43 1.89 2.37 2.32
NFATc1
180 kDa
130 kDa
1.00 0.81 1.10 1.01 0.42 0.37
RANKL
43 kDa
1.00 0.89 0.96 0.93 0.66 0.70
TGFβ
34 kDa
26 kDa
17 kDa
11 kDa
1.00 1.03 0.97 0.98 0.66 0.46
β-actin
55 kDa
43 kDa

Fig. 7C

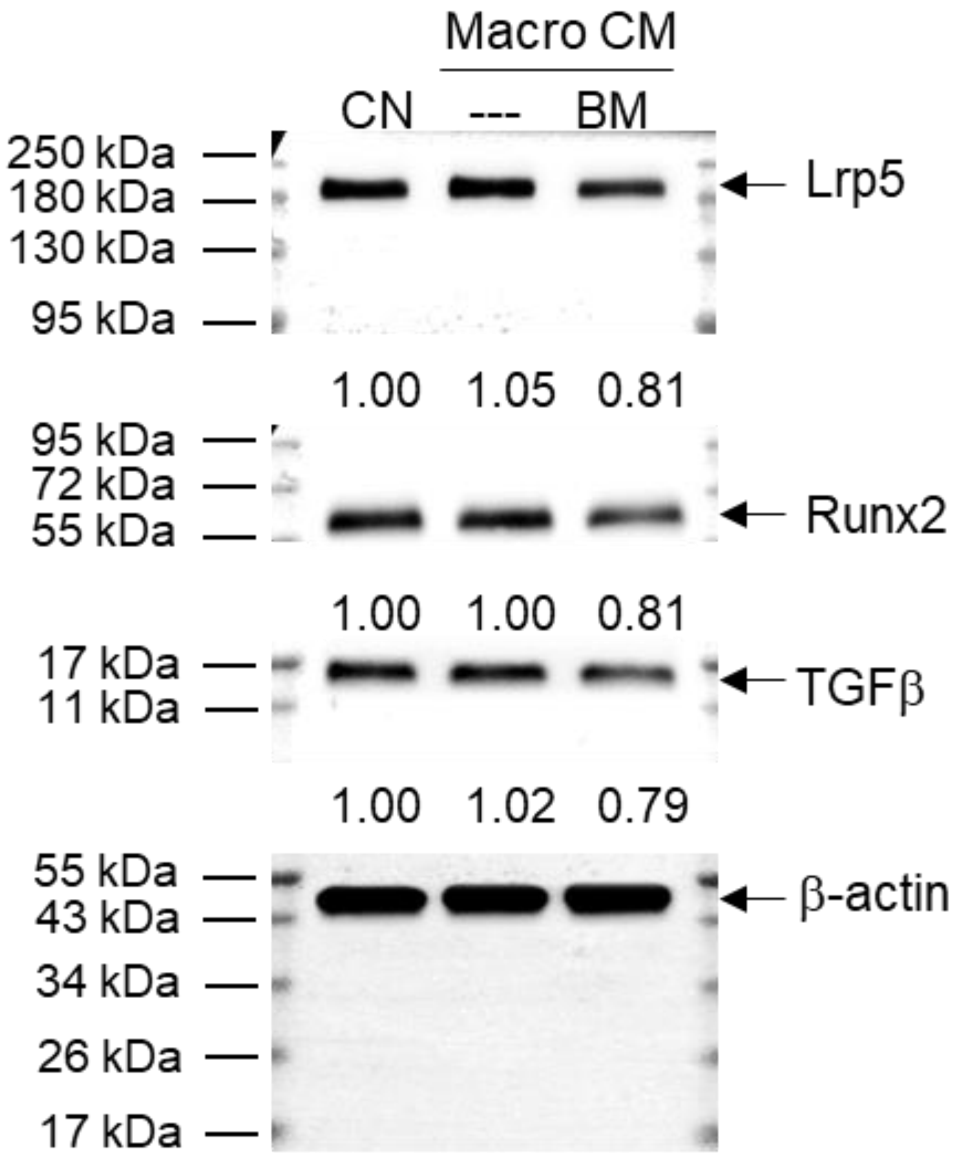
Macro CM
CN

BM
250 kDa
180 kDa
130 kDa
95 kDa
Lrp5
1.00 1.05 0.81
95 kDa
72 kDa
55 kDa
Runx2
1.00 1.00 0.81
17 kDa
11 kDa
TGFβ
1.00 1.02 0.79
55 kDa
43 kDa
34 kDa
26 kDa
17 kDa
β-actin

Fig. 7E

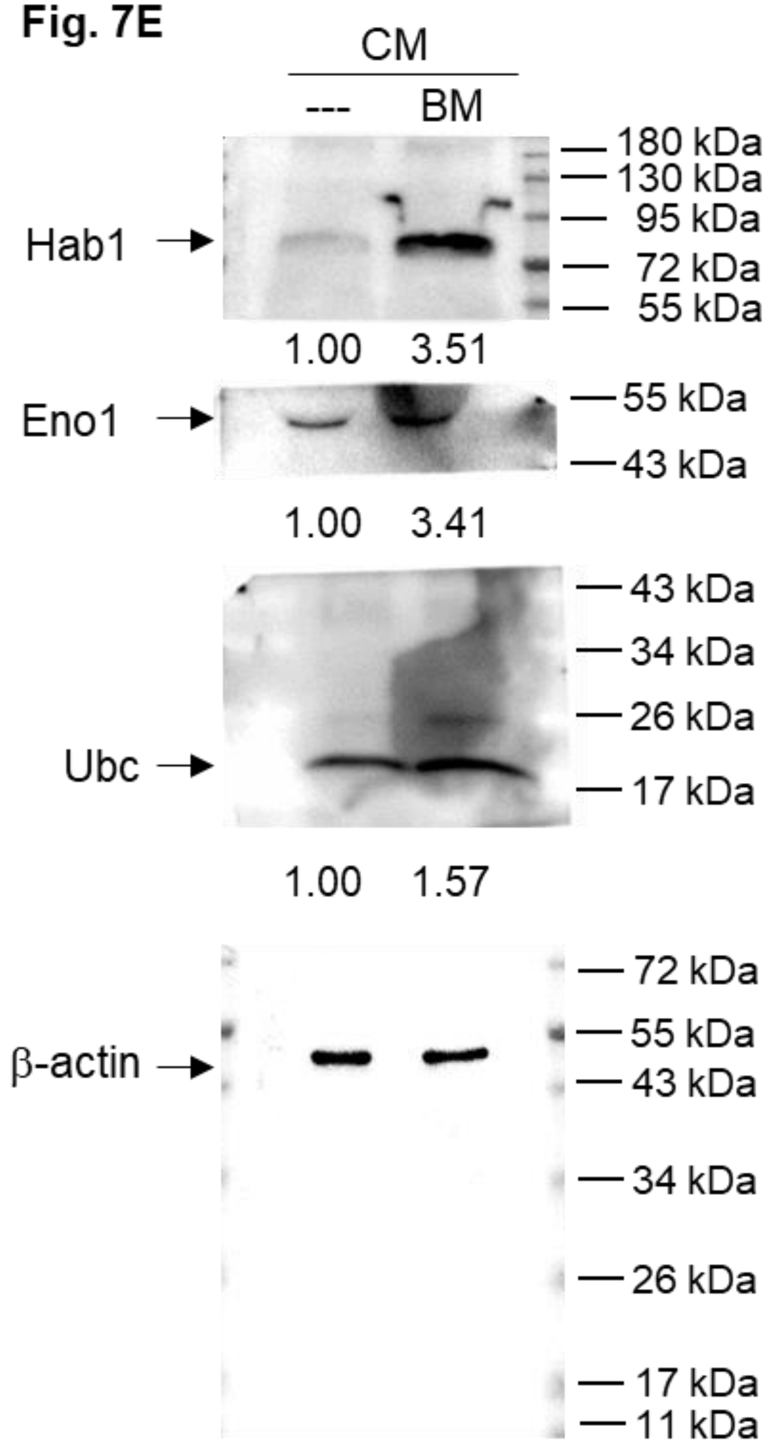

CM

BM
Hab1
180 kDa
130 kDa
95 kDa
72 kDa
55 kDa
1.00
3.51
Eno1
55 kDa
43 kDa
1.00
3.41
43 kDa
34 kDa
26 kDa
Ubc
17 kDa
1.00
1.57
72 kDa
55 kDa
β-actin
43 kDa
34 kDa
26 kDa
17 kDa
11 kDa

Fig. 8A

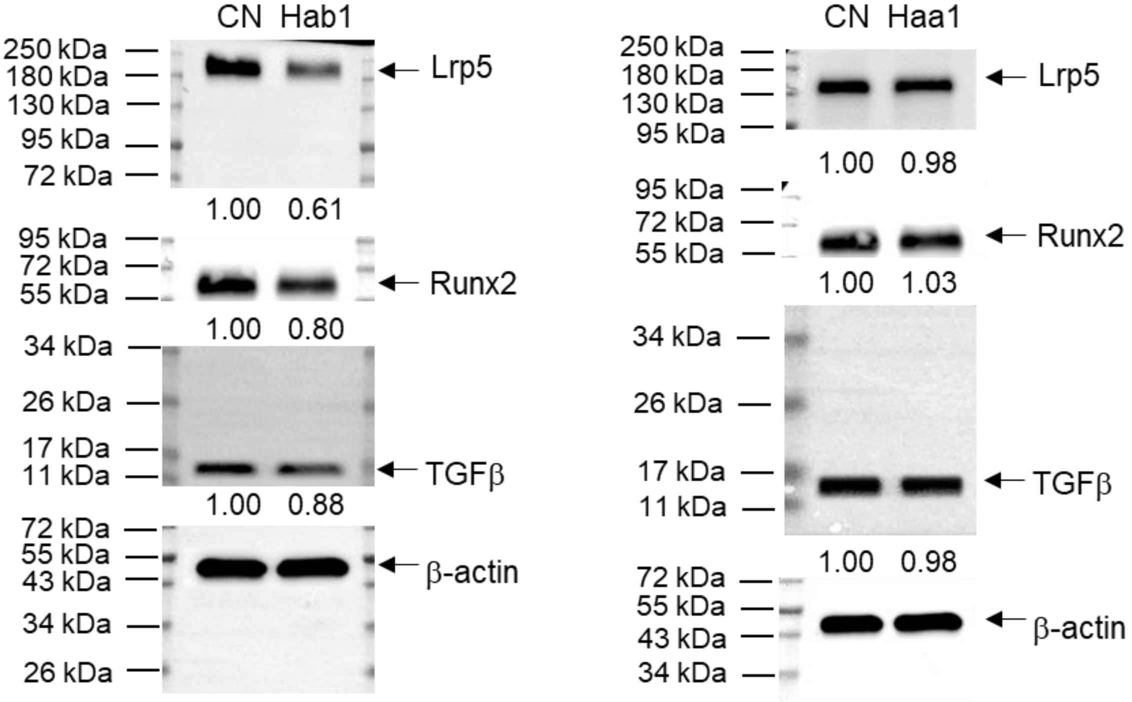

CN Hab1
250 kDa
180 kDa
130 kDa
95 kDa
72 kDa
Lrp5
1.00 0.61
95 kDa
72 kDa
55 kDa
Runx2
1.00 0.80
34 kDa
26 kDa
17 kDa
11 kDa
TGFβ
1.00 0.88
72 kDa
55 kDa
43 kDa
34 kDa
26 kDa
β-actin
CN Haa1
250 kDa
180 kDa
130 kDa
95 kDa
Lrp5
1.00 0.98
95 kDa
72 kDa
55 kDa
Runx2
1.00 1.03
34 kDa
26 kDa
17 kDa
11 kDa
TGFβ
1.00 0.98
72 kDa
55 kDa
43 kDa
34 kDa
β-actin

Fig. 8B

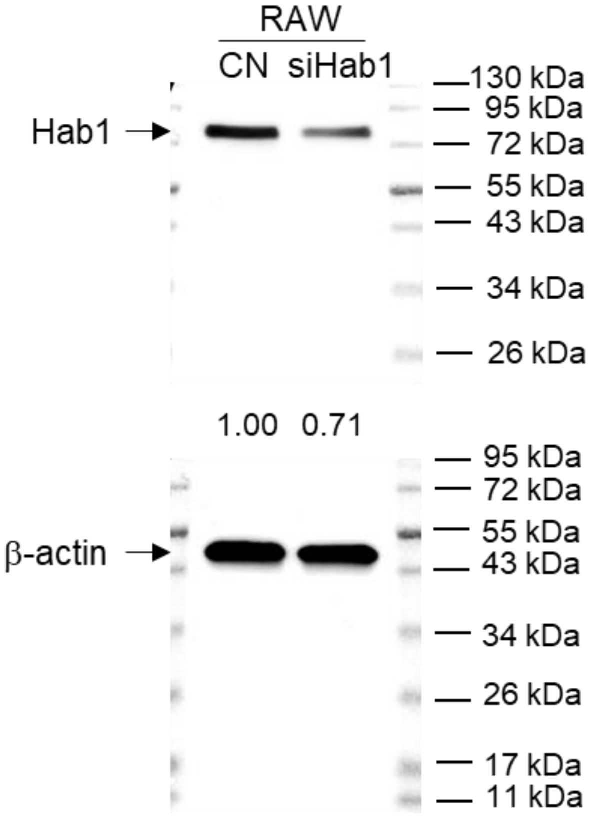

RAW
CN siHab1
Hab1
130 kDa
95 kDa
72 kDa
55 kDa
43 kDa
34 kDa
26 kDa
1.00 0.71
β-actin
95 kDa
72 kDa
55 kDa
43 kDa
34 kDa
26 kDa
17 kDa
11 kDa

Fig. 8C

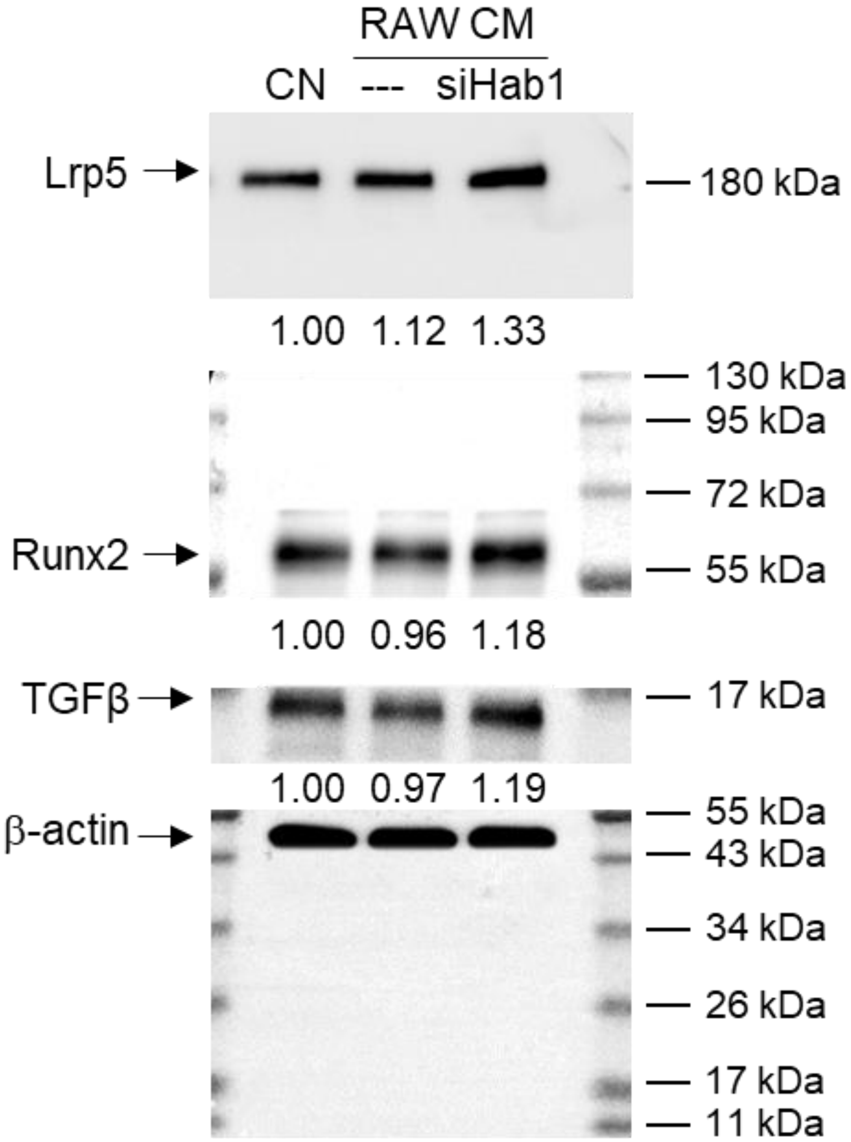
RAW CM
CN
---
siHab1
Lrp5
180 kDa
1.00 1.12 1.33
130 kDa
95 kDa
72 kDa
Runx2
55 kDa
1.00 0.96 1.18
TGFβ
17 kDa
1.00 0.97 1.19
β-actin
55 kDa
43 kDa
34 kDa
26 kDa
17 kDa
11 kDa

Fig. 8D

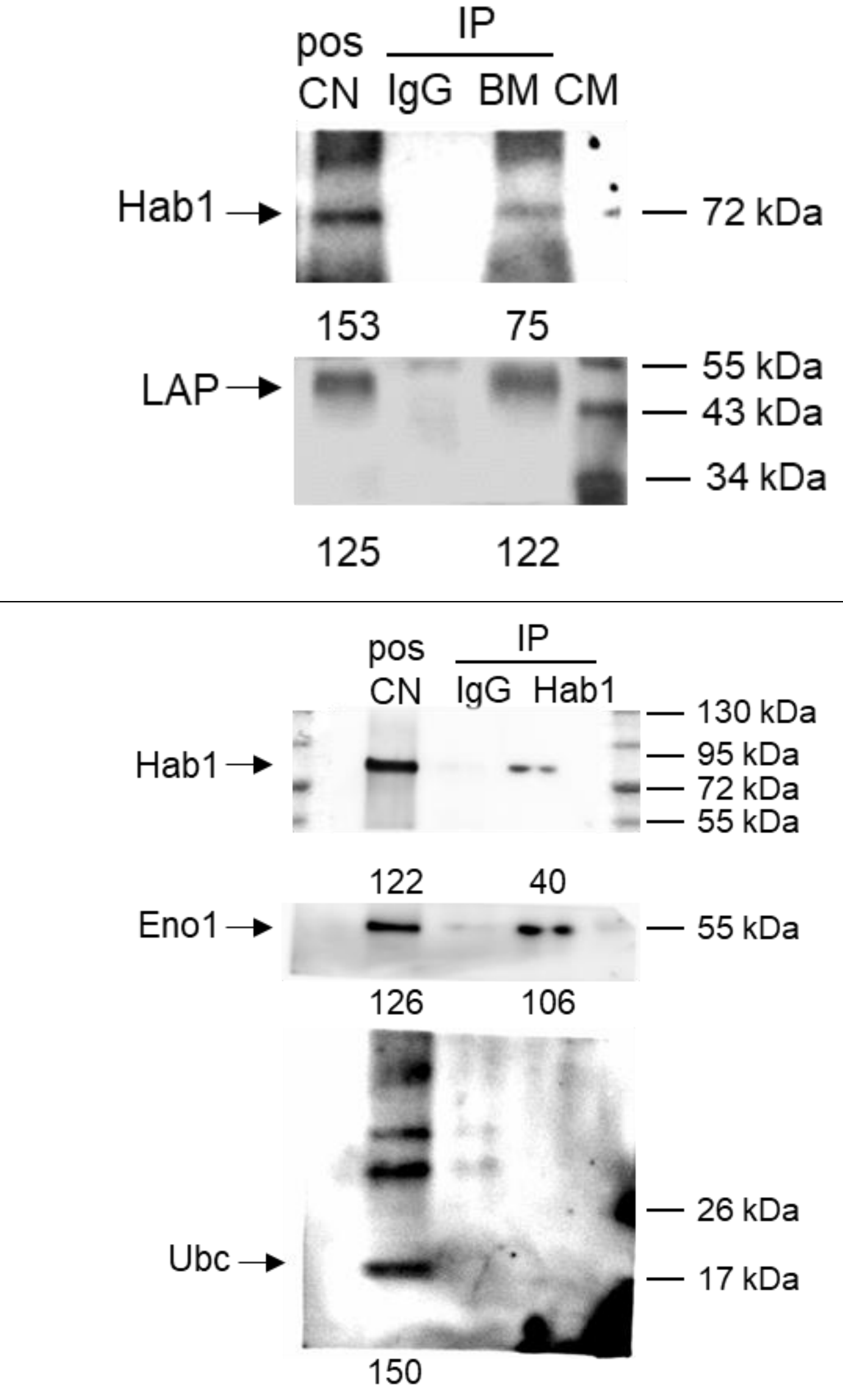
pos CN
IP
IgG
BM
CM
Hab1
72 kDa
153
75
LAP
55 kDa
43 kDa
34 kDa
125
122
pos CN
IP
IgG
Hab1
Hab1
130 kDa
95 kDa
72 kDa
55 kDa
122
40
Eno1
55 kDa
126
106
Ubc
26 kDa
17 kDa
150

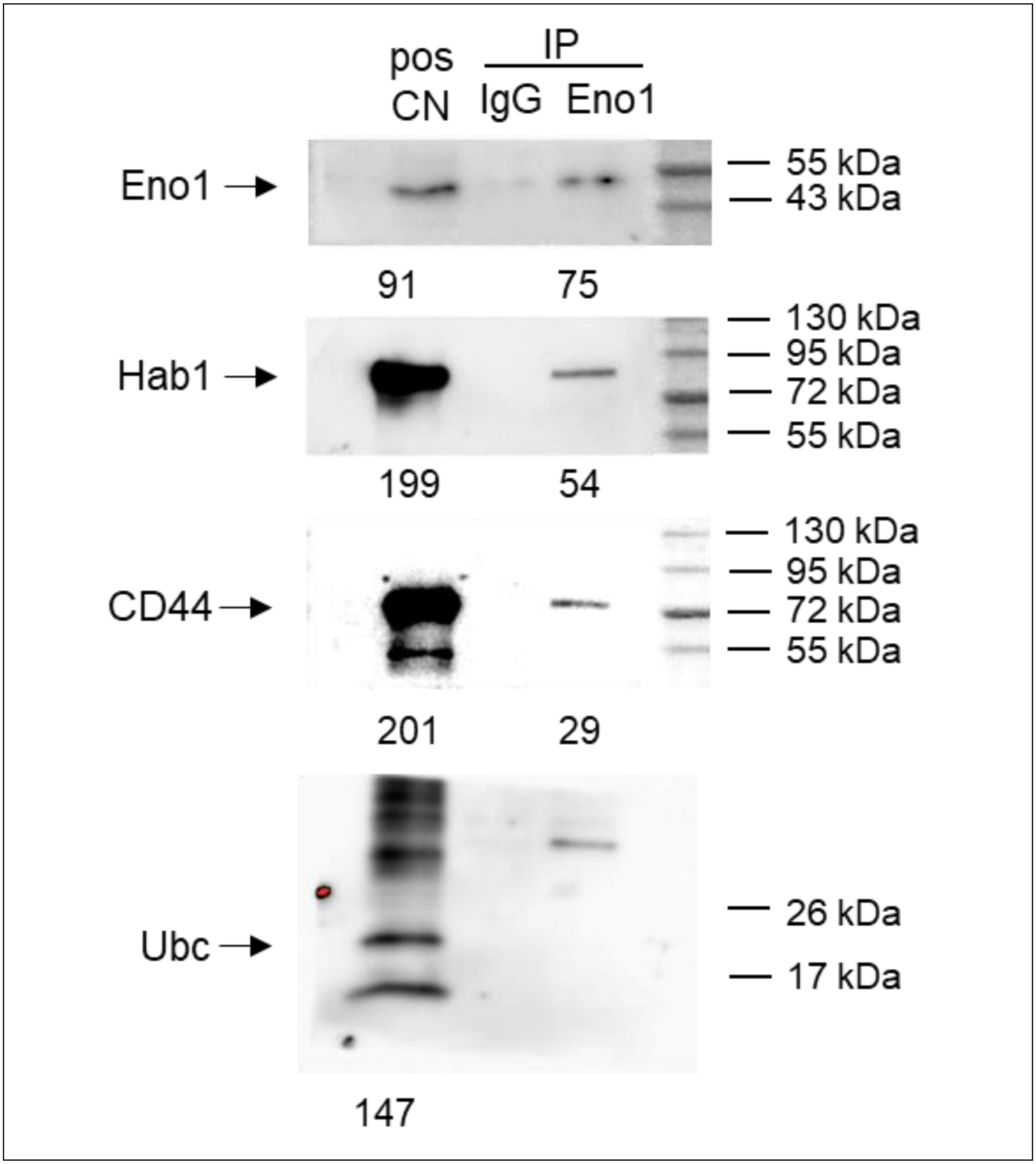
pos
CN
IP
IgG
Eno1
Eno1
55 kDa
43 kDa
91
75
Hab1
130 kDa
95 kDa
72 kDa
55 kDa
199
54
CD44
130 kDa
95 kDa
72 kDa
55 kDa
201
29
Ubc
26 kDa
17 kDa
147

Fig. 8E

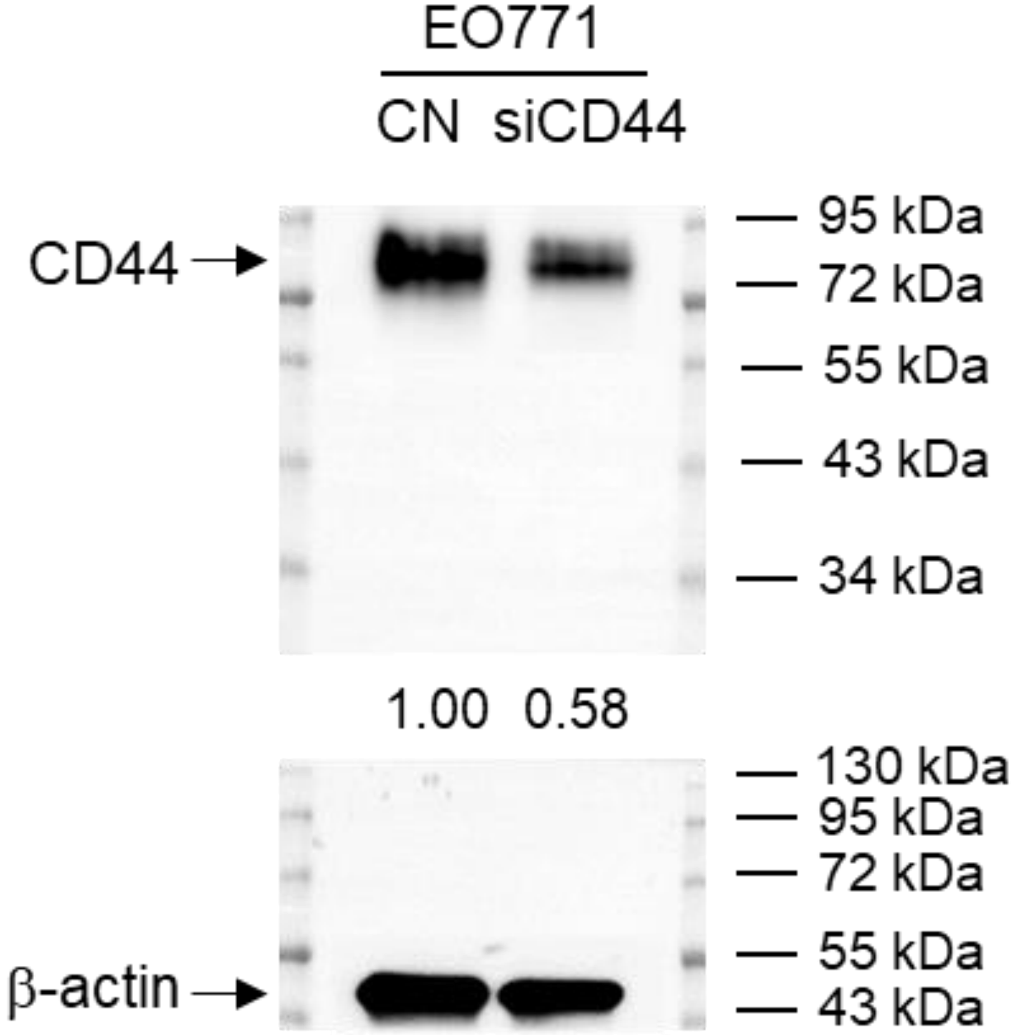
EO771
CN
siCD44
CD44
95 kDa
72 kDa
55 kDa
43 kDa
34 kDa
1.00
0.58
130 kDa
95 kDa
72 kDa
55 kDa
43 kDa
β-actin

Fig. 8G

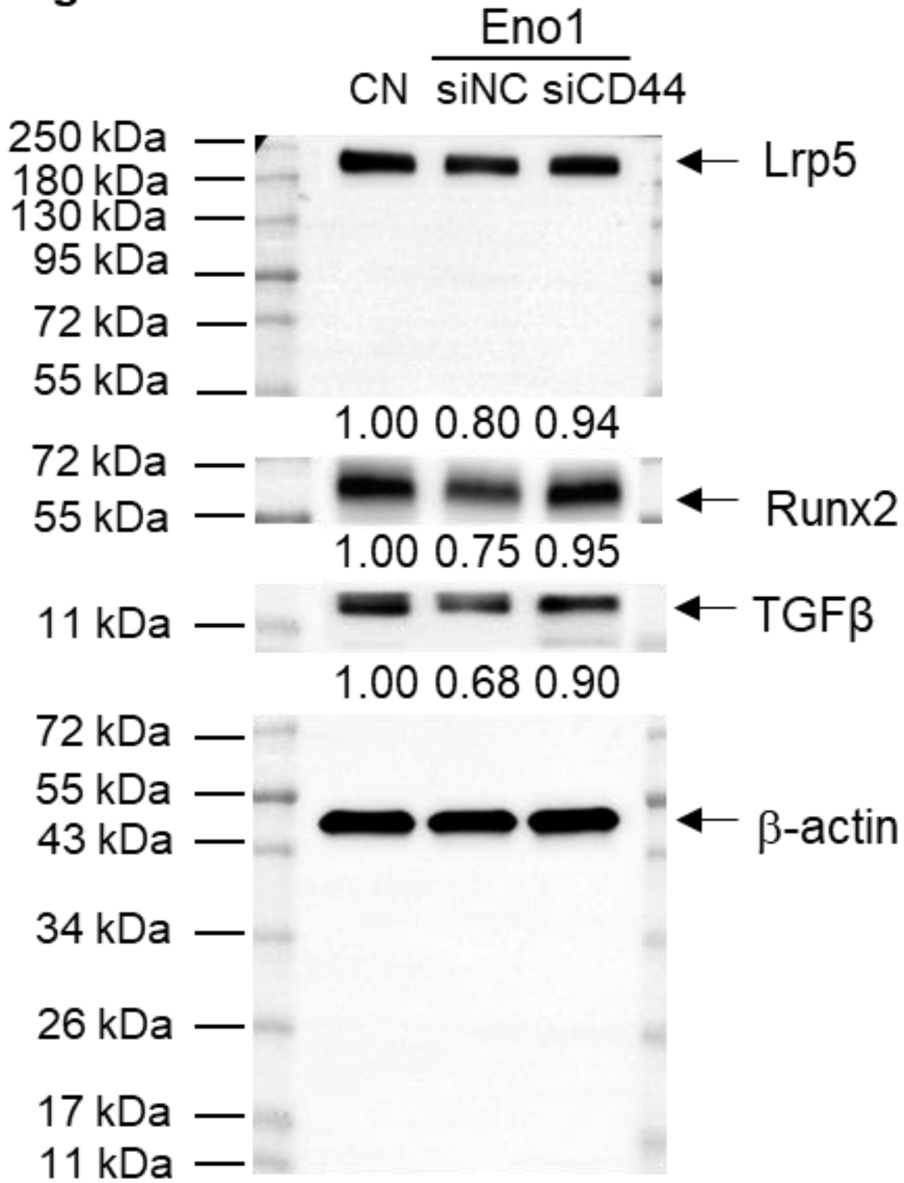
Eno1
CN
siNC
siCD44
250 kDa
180 kDa
130 kDa
95 kDa
72 kDa
55 kDa
Lrp5
1.00 0.80 0.94
72 kDa
55 kDa
Runx2
1.00 0.75 0.95
11 kDa
TGFβ
1.00 0.68 0.90
72 kDa
55 kDa
43 kDa
34 kDa
26 kDa
17 kDa
11 kDa
β-actin

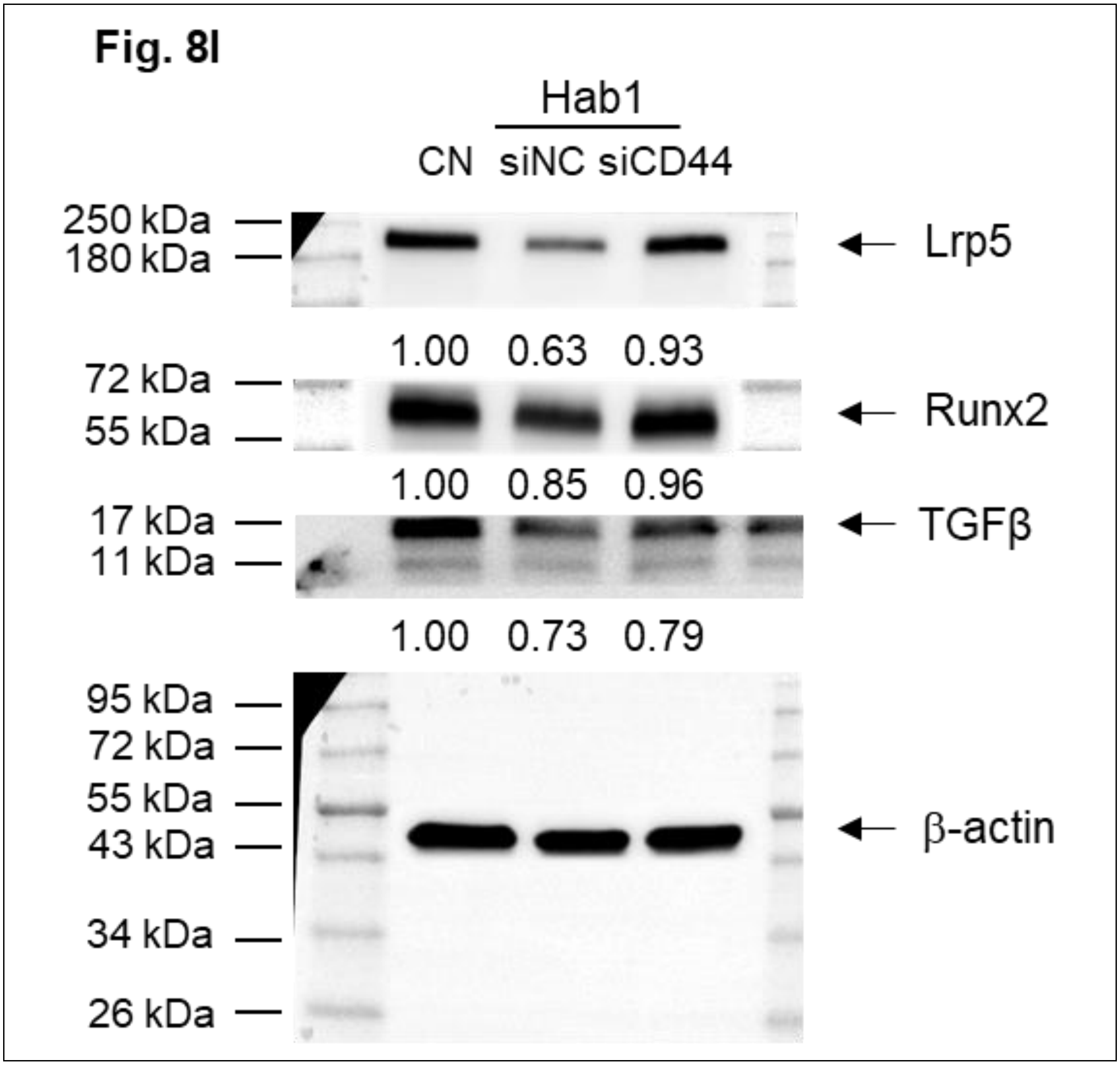
Fig. 8l
Hab1
CN
siNC
siCD44
250 kDa
180 kDa
Lrp5
1.00
0.63
0.93
72 kDa
55 kDa
Runx2
1.00
0.85
0.96
17 kDa
11 kDa
TGFβ
1.00
0.73
0.79
95 kDa
72 kDa
55 kDa
43 kDa
34 kDa
26 kDa
β-actin
